# *In Situ* Growth of Highly Adhesive Surface Layer on Titanium Foil as Durable Counter Electrodes for Efficient Dye-sensitized Solar Cells

**DOI:** 10.1038/srep34596

**Published:** 2016-10-03

**Authors:** Wantao Liu, Peng Xu, Yanjun Guo, Yuan Lin, Xiong Yin, Guangshi Tang, Meng He

**Affiliations:** 1 CAS Key Laboratory of Nanosystem and Hierarchical Fabrication, National Center for Nanoscience and Technology, Beijing, 100190, P. R. China; 2 State Key Laboratory of Chemical Resource Engineering, Department of Chemistry, School of Science, Beijing University of Chemical Technology, Beijing, 100029, P. R. China; 3 Institute of Chemistry, Chinese Academy of Sciences, Beijing, 100190, P. R. China; 4 State Key Laboratory of Chemo/Biosensing and Chemometrics, Hunan University, Changsha, 410082, P.R. China; 5 School of Physical Sciences, University of Chinese Academy of Sciences, Beijing, 100049, China

## Abstract

Counter electrodes (CEs) of dye-sensitized solar cells (DSCs) are usually fabricated by depositing catalytic materials on substrates. The poor adhesion of the catalytic material to the substrate often results in the exfoliation of catalytic materials, and then the deterioration of cell performance or even the failure of DSCs. In this study, a highly adhesive surface layer is *in situ* grown on the titanium foil via a facile process and applied as CEs for DSCs. The DSCs applying such CEs demonstrate decent power conversion efficiencies, 6.26% and 4.37% for rigid and flexible devices, respectively. The adhesion of the surface layer to the metal substrate is so strong that the photovoltaic performance of the devices is well retained even after the CEs are bended for 20 cycles and torn twice with adhesive tape. The results reported here indicate that the *in situ* growth of highly adhesive surface layers on metal substrate is a promising way to prepare durable CEs for efficient DSCs.

DSCs have attracted much attention since the advent due to the facile fabrication, potentially low-cost and competitive power conversion efficiency^1^,^2^,^3^. A typical DSC consists of three components: a dye-sensitized photo-anode, electrolyte and a counter electrode (CE)^1^,^2^,^3^. An efficient counter electrode should possess both high conductivity and superior catalytic activity on the redox couple^4^,^5^,^6^. Metal Pt is a very efficient material for the CE in DSCs, but its cost, long-term stability in triiodide ions solution, and natural scarcity hinder the mass application of DSCs^5^,^6^,^7^. As alternatives to Pt, carbon-based materials^8^,^9^,^10^,^11^, conducting polymers^12^,^13^,^14^,^15^ and inorganic metallic/semiconductive compounds^16^,^17^,^18^,^19^ have been reported. Among these materials, inorganic metallic/semiconducting compounds have been explored extensively as counter electrodes due to the ease of preparation and the excellent catalytic activity^20^.

It was reported that metal nitrides have electronic structures similar with that of Pt metal^21^,^22^. Titanium nitride has been applied as the CE for DSCs, and showed superior catalytic activity toward triiodide reduction and favourable corrosion resistance^23^,^24^. Titanium carbide (TiC) is well known for its high hardness, good electrical conductivity and excellent environmental stability^25^. Recent studies demonstrated that TiC was also efficient in catalysing triiodide reduction and could be applied as the CEs for DSCs^20^,^26^.

Most Pt-free counter electrodes are fabricated by depositing catalytic materials on substrates. Usually, the catalytic materials are deposited on substrates by doctor blade coating, dip-coating, spin coating, spraying or printing. The adhesion of catalytic materials to the substrate can be very problematic. In some cases, binders, which usually deteriorate the performance of CEs, have to be added into the catalytic materials to improve the adhesion between the substrate and catalytic layer^27^. Even so, catalytic layers are still easy to detach from the substrate, resulting in the performance deterioration or failure of DSCs. Therefore, it is strongly desired to develop durable CEs for DSCs. Unfortunately, the durability of CEs has got sufficient attention only in a very limited number of cases^28^,^29^,^30^.

Here we report the *in situ* growth of a highly adhesive surface layer on titanium foil and its application as the durable CE for efficient DSCs. In contrast to the catalytic layers which are deposited on the substrate by doctor-blading coating, dip coating or spin coating, the surface layer *in situ* grown on the titanium foil is strongly bonded to the substrate, which is evidenced by the bending and tape tests. The strong bonding between the catalytic layer and substrate, which is achieved in the *in situ* growth process, improves the durability of the CE performance greatly.

## Results and Discussion

**Figure 1:**
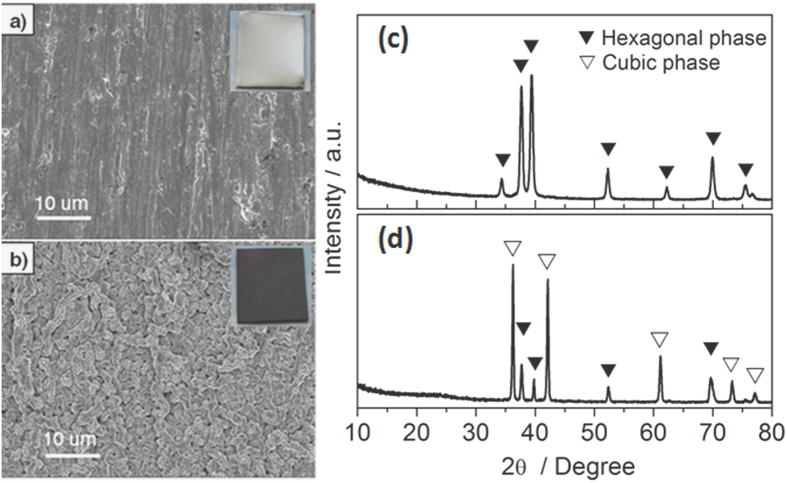
SEM images and photographs of the titanium foil before (**a**) and after (**b**) the *in situ* growth; X-ray powder diffraction patterns of the titanium foil before (**c**) and after (**d**) the growth process.

The optical photographs of the titanium foil before and after the reaction were shown as insets of Fig. 1a,b, respectively. The colour of titanium foil turned from bright white to dark brown. Both the products and the unreacted titanium foil were characterized with scanning electron microscopy. SEM images shown in Fig. 1 revealed that the surface layer of the reacted titanium foil (Fig. 1b) was distinctly different from that of the unreacted one in morphology (Fig. 1a). In contrast to the smooth surface of the unreacted titanium foil, a wrinkled rough surface was observed for the reacted titanium foil. We suppose that the wrinkled rough surface is beneficial for catalysing the triiodide reduction due to more active sites available compared with the smooth surface. Cross-sectional SEM images (not shown) revealed the fluctuant profile of the surface but no obvious stratification was observed between the surface and matrix.

Shown in Fig. 1c and d are X-ray diffraction (XRD) patterns of the titanium foil before and after the *in situ* growth process, respectively. All reflections of the unreacted foil can be attributed to hexagonal close-packed Ti metal with lattice parameters *a* = 3.015(2) Å and *c* = 4.700(2) Å^31^. Reflections resulting from Ti metal were also observed on the XRD pattern of the reacted foil. Besides reflections of Ti metal, other reflections of the reacted foil can be assigned to a face-centred cubic phase with lattice parameter a = 4.283(2) Å. The face-centred cubic phase is consistent with TiN and TiC. The lattice parameters of TiN and TiC were reported to be a = 4.2410 Å^32^ and a = 4.32467 Å^33^, respectively. The lattice parameter observed for the cubic phase in this study lies in between the above two values, indicating that the cubic phase may be Ti(C, N), possibly with a homogeneity range. In addition, a non-significant hump was observed at the low angle of this powder pattern, indicating the possible presence of amorphous phase in the products.

**Figure 2:**
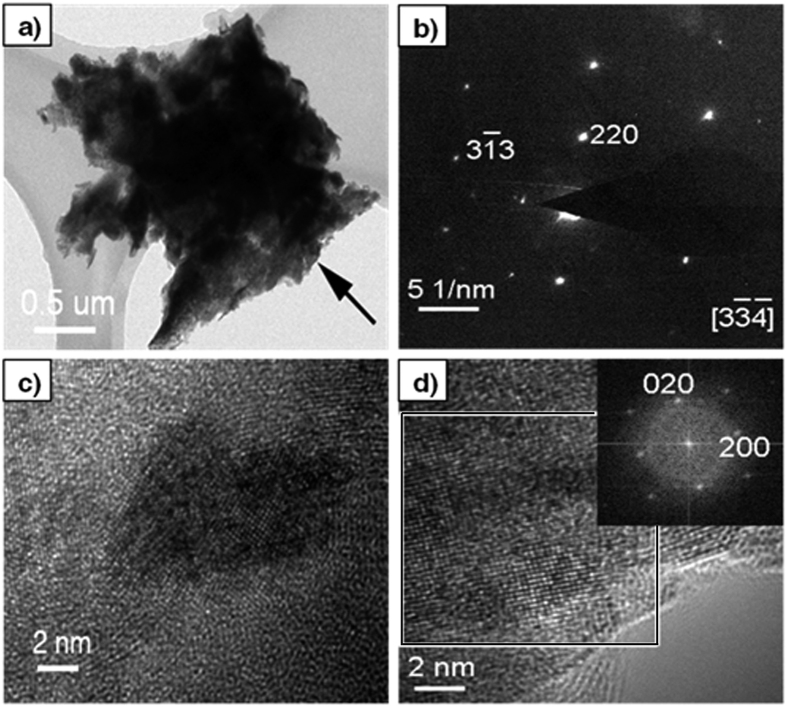
(**a**) TEM image of an exfoliation peeled off with a scalpel from the surface of the reacted Ti foil. (**b**) SAED pattern taken from the edge area which is pointed with an arrow in (**a**). HRTEM images taken from different region of the exfoliation are shown in (**c**) and (**d**). The inset in (**d**) is the Fourier transform (FT) of the region marked with a square.

The surface of the reacted titanium foil was scratched with a scalpel, and the exfoliations were investigated with transmission electron microscopy. The low magnification TEM image of an exfoliation was shown in Fig. 2a. The selected area electron diffraction (SAED) pattern shown in Fig. 2b was taken from the edge area marked with an arrow in Fig. 2a. The SAED pattern can be indexed with lattice parameters of the face-centred cubic Ti(C, N), which is identified by XRD. Shown in Fig. 2c is a high-resolution TEM (HRTEM) image which reveals the presence of amorphous phase in the products in addition to the crystalline phase. The Fourier transform of a crystalline region marked with a square in Fig. 2d is shown as an inset, which agrees well with the cubic unit cell of Ti(C, N). This further confirms that the crystallites in the products are cubic Ti(C, N).

**Figure 3:**
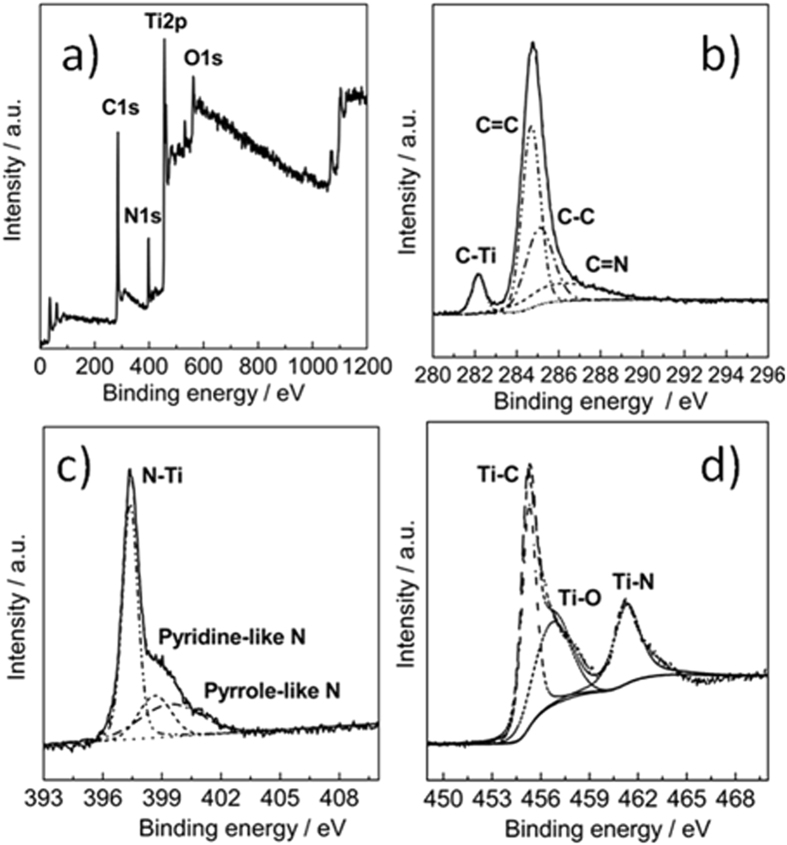
XPS survey spectrum (**a**) and core level spectra of C 1s (**b**), N 1s (**c**) and Ti 2p (**d**) of the surface layer grown on titanium foil.

The reacted Ti foil was also characterized with X-ray photoelectron spectroscopy (XPS). The survey spectrum was given in Fig. 3a. As expected, Ti, C and N were detected from the surface. In addition, O was also detected, which might result from the residual oxide overlayer of Ti substrate or the leakage of the growth system. Shown in Fig. 3b is the core level spectrum of C 1s. It can be disintegrated into four subpeaks located at 284.7, 285.1, 286.1 and 282.0 eV respectively. The strong subpeaks at 284.7 and 285.1 eV are attributed to graphite-like sp^2^ C and sp^3^ C. The subpeak centred at 286.1 eV is corresponded to nitrogenated carbon (C=N). The subpeak located at 282.0 eV results from carbon bonded to Ti (C-Ti)^34^. The core level spectrum of N 1s (shown in Fig. 3c) can be deconvoluted into three subpeaks. The subpeak centered at 397.3 eV is attributed to N bonded to Ti (N-Ti), while subpeaks at 398.7 and 399.6 eV are signals of pyridine-like and pyrrole-like N, respectively. Core level spectrum of Ti 2p (Fig. 3d) are well fitted with three subpeaks. The subpeak with the lowest binding energy (455.0 eV) results from Ti bonded to carbon (Ti-C), while the subpeak centred at the highest binding energy is attributed to Ti bonded to nitrogen (Ti-N)^35^. The subpeak centered at 456.8 eV is the signal of oxygenated Ti (Ti-O). Combining the results of XPS measurements with those of XRD and TEM characterizations, we can draw the conclusion that a surface layer consisting of cubic Ti(C, N) crystallites and the amorphous phase of N-doped carbon formed on the titanium substrate in the process of *in situ* growth. Moreover, carbon, nitrogen and oxygen might have been diffused into the lattice of the titanium metal close to the surface.

**Figure 4:**
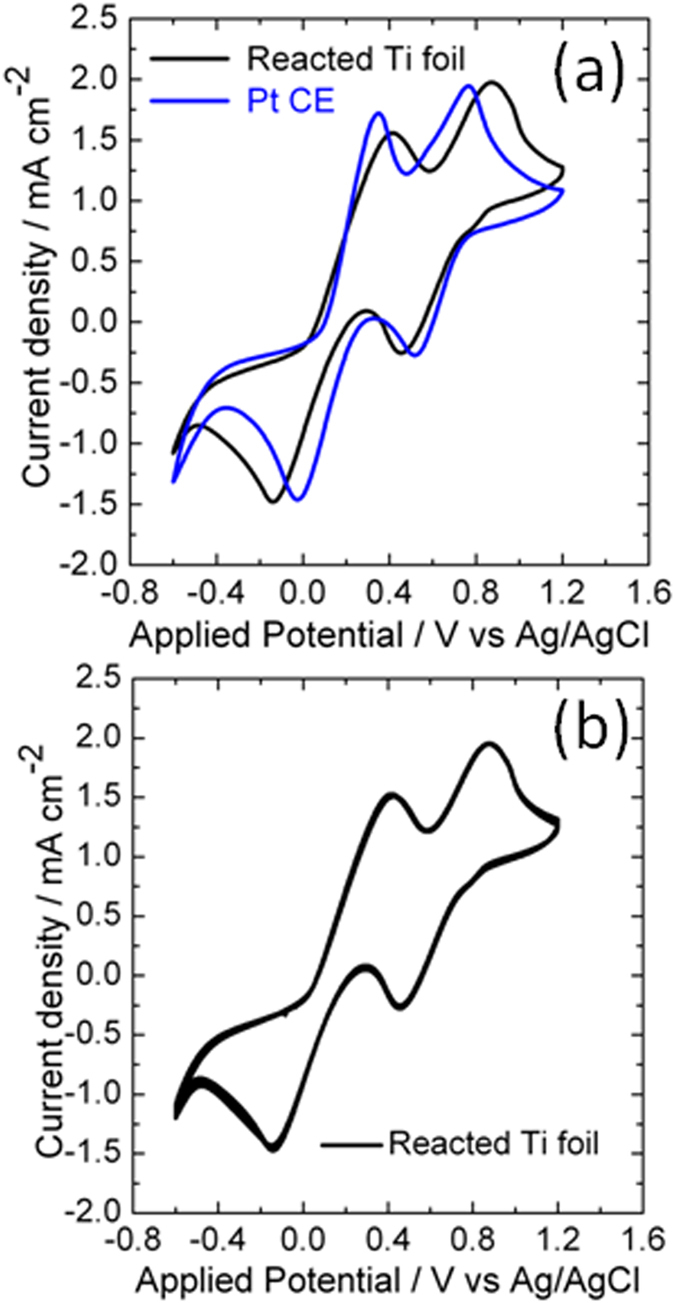
(**a**) Cyclic voltammograms of the reacted Ti foil and Pt electrodes measured at a scan rate of 50 mV s^−1^. (**b**) Cyclic voltammograms of the reacted Ti foil measured in consecutive 10 cycles at a scan rate of 50 mV s^−1^.

The catalytic activity of the reacted titanium foil towards triiodide reduction was evaluated with cyclic voltammetry and compared with that of Pt electrode. Cyclic voltammograms of both the reacted titanium foil and Pt electrode were presented in Fig. 4a. The anodic peak at relatively negative potentials is due to the triiodide reduction. As shown in Fig. 4a, the reacted titanium foil exhibited a peak reduction current density of 1.5 mA cm^−2^, which is comparable to the corresponding value of Pt electrode. This indicates that the reacted titanium foil is as efficient as Pt CE in catalysing the reduction of triiodide^36^,^37^. In addition, cyclic voltammograms for both the starting Ti foil and the treated Ti foil in the absence of melamine were also conducted. The resultant CV curves are shown in Supplementary Information (Figure SI). No typical peaks for triiodide reduction process are found in the cases of the two electrodes. These results imply that the high catalytic performance of the reacted titanium foil is due to the treatment of Ti foil with melamine. Plots shown in Fig. 4b are the CV curves of consecutive 10 cycles measured on the reacted titanium foil. All the curves coincide with each other very well, indicating the good electrochemical stability of the reacted titanium foil^36^,^37^.

**Figure 5:**
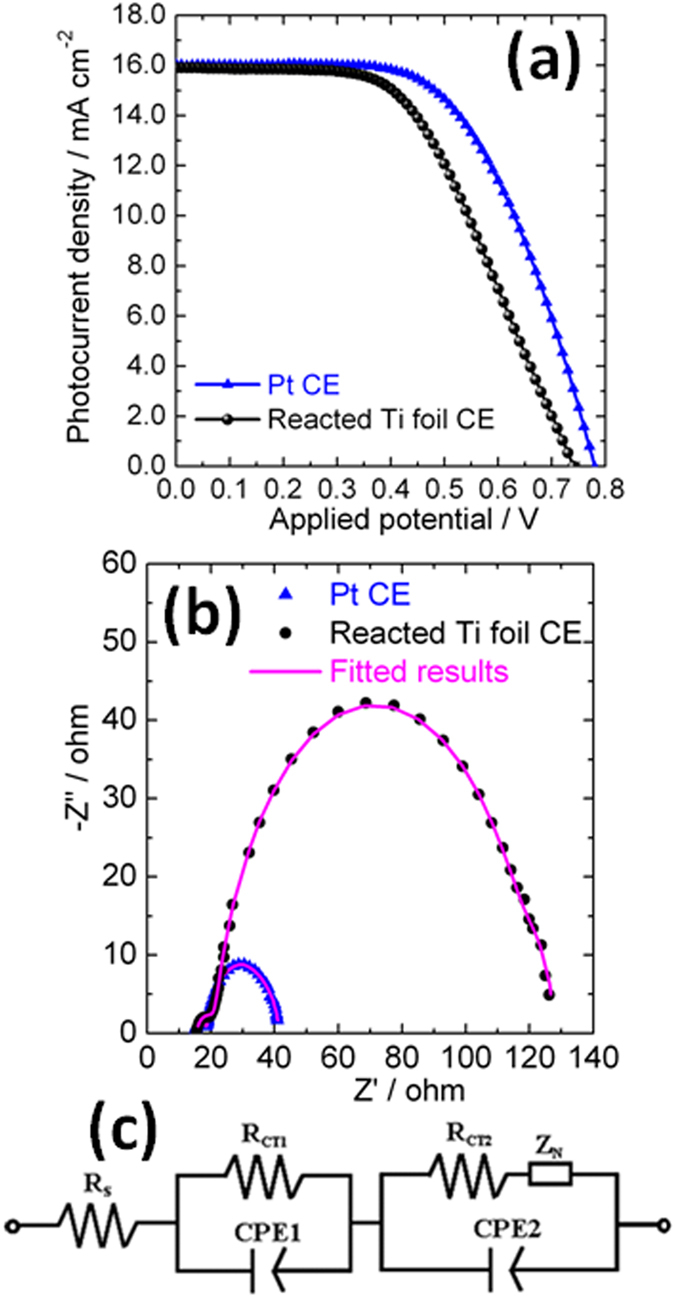
(**a**) Current density−voltage (J−V) curves of the rigid DSCs with a Pt CE and a reacted Ti foil CE; (**b**) Electrochemical impedance spectra of DSCs using Pt (blue solid triangle) and the reacted Ti foil (black solid circle) counter electrodes measured under the illumination of simulated solar light (AM 1.5 G, 100 mW cm^−2^) and pink solid lines present the corresponding fitted results; (**c**) The fitting equivalent circuit used in the study.

Both the rigid and flexible DSCs were fabricated using the reacted Ti foils as the CEs. For comparison, the corresponding devices applying conventional Pt CEs were also prepared as reference. The photocurrent density-voltage (J-V) curves of rigid devices are given in Fig. 5a. The DSC applying the reacted titanium foil as the CE exhibited a short-circuit current density, J_sc_, of 15.90 mA cm^−2^, which is comparable to that of the device based on the Pt CE (16.03 mA cm^−2^). However, lower open-circuit voltage, V_oc_, and fill factor (FF) were observed for the DSC containing the reacted titanium foil in comparison with the device using Pt CE. The PCE of the device based on the reacted Ti foil is 6.26%, about 85% of the PCE exhibited by the DSC containing Pt CE. The electrochemical impedance spectra (EIS) of rigid DSCs were measured under the illumination of simulated AM 1.5 G solar light (100 mW cm^−2^), and the corresponding Nyquist plots were presented in Fig. 5b. Two semicircles were observed on the each Nyquist plot. The high-frequency arc is due to the charge-transfer resistance at the interface of CE/electrolyte (R_CT1_), whereas the arc appearing at lower frequency is ascribed to the charge transfer resistance at the interface of photo-anode/electrolyte (R_CT2_) and diffusion resistance of I^−^/I_3_^−^ in the electrolyte (Z_N_). The onset of the high frequency arc is determined by the substrate resistance R_S_^38^,^39^,^40^,^41^. The Nyquist plots are fitted using the equivalent circuit shown in Fig. 5c. The fitted values of R_S_, R_CT1_, R_CT2_ and Z_N_ of both devices are summarized in Table 1. The two devices presented similar R_S_ values (14.95 Ω for Pt-based cell, 15.37 Ω for the reacted Ti foil based device). Small values of R_CT1_ were observed for both devices using the reacted Ti foil and Pt CEs, further confirming that both CEs were efficient in catalysing the reduction of tri-iodide. However, a much larger R_CT2_, 99.46 Ω, was observed for the DSC using a reacted Ti foil CE, in comparison with the corresponding value of the device using a Pt CE, 20.95 Ω. This is thought to be the reason for the slightly lower fill factor exhibited by the DSC with the reacted Ti foil CE^38^,^39^,^40^,^41^.

**Figure 6:**
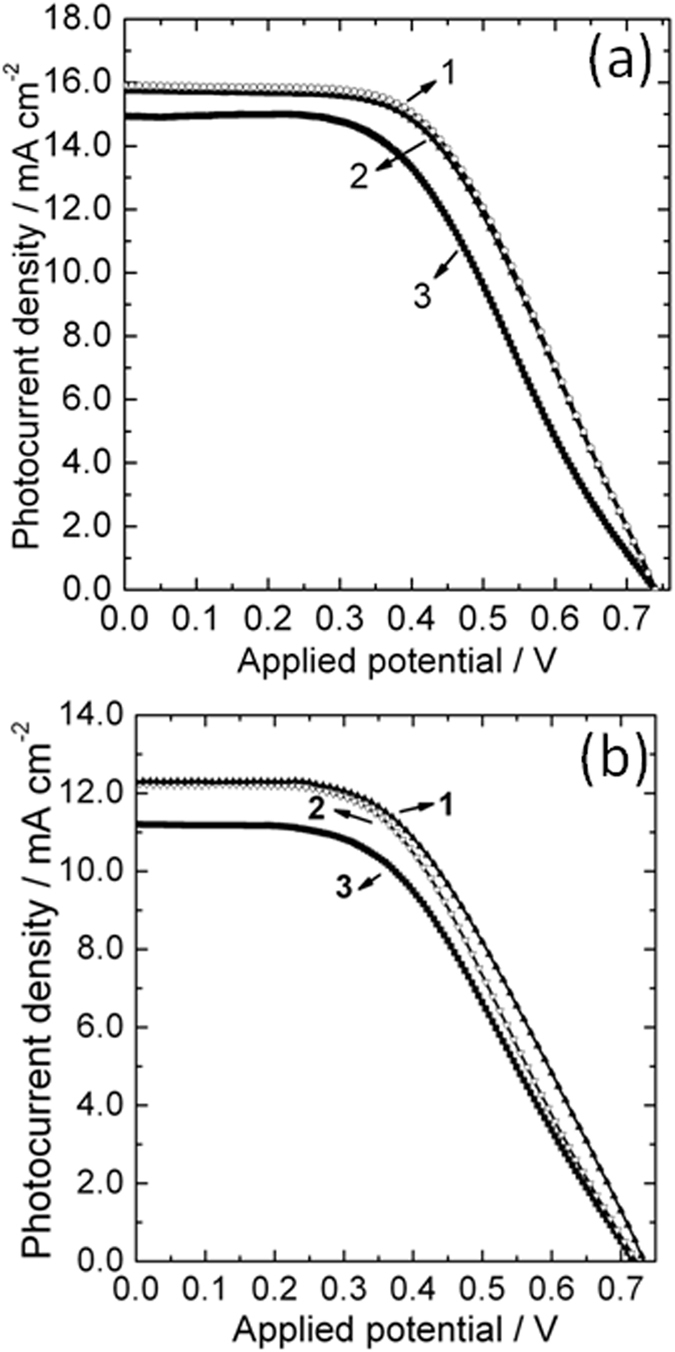
Current density−voltage (J−V) curves of the rigid (**a**) and flexible (**b**) DSCs. Curves 1, 2 and 3 correspond to the devices using the reacted Ti foil, the reacted Ti foil after bending tests and the reacted Ti foil after tape tests as counter electrodes, respectively.

The reacted titanium foil is still ductile and bendable. It was bended back and forth with a bending angle up to about 60° for 20 cycles, and then assembled into a DSC again as the CE. The J-V curves measured before and after the bending cycles are presented in Fig. 6a. As demonstrated by Fig. 6a, twenty bending cycles only led to very little drop in the photovoltaic performance of the DSC containing the reacted titanium foil CE. The adhesion of the surface layer to the metal matrix was further examined by tape tests. A Scotch tape was pressed on the surface of the reacted Ti foil with hand, and then peeled off from the surface. In the peeling process, the tape is kept to be perpendicular to the surface of the Ti foil. This procedure was repeated twice before the reacted Ti foil was assembled into the DSC again. A PCE of 5.27% was exhibited by such a device, about 84% of its initial efficiency, and the corresponding photovoltaic parameters are listed in Table 2. A flexible DSC was fabricated using a plastic TiO_2_ photoanode and the reacted Ti foil as the CE. Bending and tape tests were also made on the CE of the flexible device. Photovoltaic performance of the flexible device drops only slightly after the bending and tape tests, as evidenced by the J-V curves shown in Fig. 6b. The detailed photovoltaic parameters of the flexible devices were also summarized in Table 2. The results of bending and tape tests demonstrated solid evidence for the strong adhesion of the catalytic surface layer to the Ti metal matrix. The strong adhesion between the catalytic layer and the substrate is essential for a durable CE. Though many efficient Pt-free CEs have been reported, the durability of CEs has not been paid adequate attention. Actually, the durability is as important as the efficiency for the practical application of DSCs. Compared with spin coating, dip coating and/or spraying techniques, *in situ* growth seems to be more promising in preparing durable CEs, and warrants more attention in the exploration for new alternatives to the conventional Pt CE.

## Conclusion

In summary, a highly adhesive surface layer has been prepared on the titanium foil via a facile *in situ* growth process using melamine as C and N sources. The surface layer consisted of cubic Ti(C, N) crystallites and amorphous N-doped carbon. The titanium foil covered with the surface layer exhibited superior catalytic activity on the reduction of triiodide, and was applied as the CEs for both the rigid and flexible DSCs. The surface layer was so strongly bonded to the metal matrix that it was resistant to the bending and tape tests. The resistance to bending and tape-tearing benefits the durability of CEs, which is essential for the practical application of DSCs. The durability of CEs deserves more attention in the development of alternatives to Pt CE, and the *in situ* growth of catalytic surface layer on substrates seems to be a promising way of preparing high-performance durable CEs.

## Methods

### *In situ* growth of surface layer on Ti foil

The experimental setup for the *in situ* growth consists mainly of a tube furnace equipped with a quartz tube of 1 inch in diameter and a flow-meter to control the rate of the carrying gas. In a typical synthetic run, a piece of Ti foil was loaded at the center of a tube furnace, while melamine powder was positioned at an appropriate upstream place which was beyond the heating zone of the tube furnace. Before the growth, the system was purged with flowing argon at the rate of 100 sccm. After purging, the Ti foil was heated to 950 °C at a rate of 30 °C min^−1^ in the flowing argon at a rate of 100 sccm, and then held at that temperature for 1 hour. During this process, melamine was heated to about 300 °C, and sublimated/decomposed to release vapour containing N, C and H, which was delivered by the carrying gas to react with the Ti foil. The power of the furnace was turned off when the holding time was over, and the system was allowed to cool down to room temperature naturally in the flowing argon. The entire growth system was placed in the fume hood, and the growth was performed under the continuous ventilation.

### Fabrication of DSCs

A 12 μm thick TiO_2_ (20 nm) layer and 4 μm thick TiO_2_ (200 nm) scattering layer were fabricated on the FTO glass by doctor-blading technique. Then the TiO_2_ electrode was sintered at 450 °C for 30 minutes. Subsequently, 40 mM TiCl_4_ solution treatment was conducted at 70 °C for 30 minutes. After that, the TiO_2_ electrodes were sintered at 450 °C for another 30 minutes. After the temperature dropped to 85 °C, the electrodes were immersed into a mixture solution of acetonitrile and tertiary-butanol with the volume ratio of 1:1 containing 30 mM N719 dye for 12 h.

The sandwich-type cells were fabricated by assembling photo-anodes and counter electrodes with the liquid electrolyte being injected between them. The liquid electrolyte was composed of 0.1 mol L^−1^ iodine, 0.6 mol L^−1^ methylhexylimidazolium iodide, 0.5 mol L^−1^ tert-butylpyridine, and 0.1 mol L^−1^ lithium iodide in 3-methoxypropionitrile. The flexible TiO_2_ photo-anodes were prepared using electrophoretic deposition technique, as reported in our previous publication^42^. The assembly of the flexible DSCs is similar to that of the rigid ones.

### Characterization

The surface morphology of the reacted Ti foil was characterized using a field emission scanning electron microscope (FESEM, Hitachi S-4800). TEM observation was carried out with a microscope (FEI Tecnai F20 G^2^ U-twin) operated at 200 kV. X-ray powder diffraction pattern was recorded with a D/MAX TTRIII (CBO) diffractometer using Cu Kα radiation. X-ray photoelectron spectroscopy (XPS) measurements were performed with an ESCA Lab 250xi spectrometer using Al Kα (1486.6 eV) irradiation as X-ray source. All the spectra were calibrated to the binding energy of the adventitious C 1s peak at 284.8 eV. Cyclic voltammetry (CV) was carried out using CHI630D electrochemical analyzer in a three-electrode system in an acetonitrile solution composing of 0.1 mol L^−1^ LiClO_4_, 0.01 mol L^−1^ LiI and 1 × 10^−3^ mol L^−1^ I_2_. In the three-electrode system, the reacted Ti foil or Pt electrode was used as the working electrode, a platinum wire as the counter electrode, and an Ag/AgCl electrode as a reference electrode. The current density−voltage (J-V) characteristics of the DSCs were measured under AM 1.5G illumination (100 mW cm^−2^) using a solar simulator (Oriel Newport) equipped with a 150 W xenon lamp and a digital source meter (2420, Keithley Instruments, USA). The effective irradiated area was 0.2 cm^2^. Electrochemical impedance spectroscopy (EIS) measurement was carried out using a frequency response analyzer (Solartron SI 1270) and a potentiostat (Solartron 1287) at amplitude of 10 mV and the open-circuit voltage under light irradiation of 100 mW cm^−2^ in the frequency range from 0.1 to 10^5^ Hz. The EIS data was fitted using ZView software.

## Additional Information

**How to cite this article**: Liu, W. *et al. In Situ* Growth of Highly Adhesive Surface Layer on Titanium Foil as Durable Counter Electrodes for Efficient Dye-sensitized Solar Cells. *Sci. Rep.* **6**, 34596; doi: 10.1038/srep34596 (2016).

## Supplementary Material

Supplementary Information

## Figures and Tables

**Table 1:** Photovoltaic parameters and EIS fitting parameters of the rigid DSCs with Pt and reacted Ti foil CEs measured under simulated solar illumination of 100 mW cm^−2^ AM 1.5 G.

Counter Electrode	V_OC_/V	J_SC_/mA cm^−2^	FF	PCE/%	R_S/_Ω	R_CT1/_Ω	R_CT2/_Ω	Z_N/_Ω
Pt	0.778	16.03	0.59	7.36	14.95	3.46	20.95	1.82
Reacted Ti foil	0.735	15.90	0.53	6.26	15.37	5.89	99.46	6.79

**Table 2:** Photovoltaic performance of the rigid (R) and flexible (F) DSCs using the reacted titanium foil as CEs.

Counter electrode^a^	V_OC_/V	J_SC_/mA cm^−2^	FF	PCE/%
untreated (R)	0.735	15.90	0.53	6.26
Bended (R)	0.735	15.73	0.53	6.14
Torn (R)	0.736	14.92	0.48	5.27
untreated (F)	0.725	12.29	0.49	4.37
Bended (F)	0.715	12.23	0.48	4.20
Torn (F)	0.715	11.20	0.47	3.80

^a^R: rigid DSCs; F: flexible DSCs; untreated: the reacted Ti foil without further treatment; Bended: the reacted Ti foil was bended back and forth for 20 cycles; Torn: the reacted Ti foil was torn twice with Scotch tape.
