# Supplementary material for: In Situ Growth of Highly Adhesive Surface Layer on Titanium Foil as Durable Counter Electrodes for Efficient Dye-sensitized Solar Cells
Source: Sci Rep. 2016 Oct 3;6:34596. doi: 10.1038/srep34596 (PMC5046124; doi:10.1038/srep34596)
Supplement: Supplementary Information [file srep34596-s1.docx]

**Supplementary Information**

*In Situ* Growth of Highly Adhesive Surface Layer on Titanium Foil as Durable Counter Electrodes for Efficient Dye-sensitized Solar Cells

*Wantao Liu ^1,2^, Peng Xu ^1^, Yanjun Guo ^1^, Yuan Lin ^3^, Xiong Yin ^1,2, 4, *^, Guangshi Tang ^2, *^, Meng He ^1,5, *^*

*^1^ CAS Key Laboratory of Nanosystem and Hierarchical Fabrication, National Center for Nanoscience and Technology, Beijing, 100190, P. R. China.*

*^2^ State Key Laboratory of Chemical Resource Engineering, Department of Chemistry, School of Science, Beijing University of Chemical Technology, Beijing 100029, P. R. China.*

*^3^ Institute of Chemistry, Chinese Academy of Sciences, Beijing 100190, P. R. China.*

*^4^ State Key Laboratory of Chemo/Biosensing and Chemometrics, Hunan University, Changsha 410082, P.R. China*

*^5^ School of Physical Sciences, University of Chinese Academy of Sciences, Beijing 100049, China*

^*^ Corresponding authors: Dr. Xiong Yin, Email: [yinx@nanoctr.cn](mailto:yinx@nanoctr.cn); Dr. Guangshi Tang, E-mail: [tanggs@mail.buct.edu.cn](mailto:tanggs@mail.buct.edu.cn); Prof. Meng He, Email: [mhe@nanoctr.cn](mailto:mhe@nanoctr.cn) TEL: +86-10-8254-5555, Fax: +86-10-62656765.


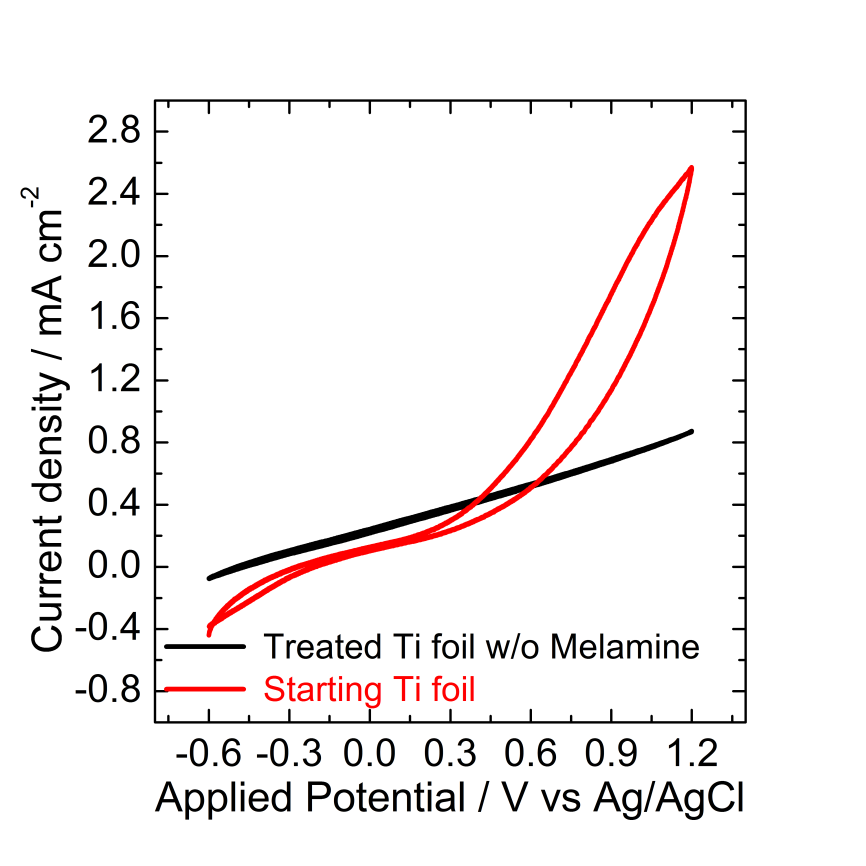


Figure SI. Cyclic voltammograms of starting Ti foil and the treated Ti foil in the absence of melamine measured at a scan rate of 50 mV s^−1^.
